# Persistent Iron Within the Infarct Core After ST-Segment Elevation Myocardial Infarction: Implications for Left Ventricular Remodeling and Health Outcomes

**DOI:** 10.1016/j.jcmg.2017.08.027

**Published:** 2018-09

**Authors:** Jaclyn Carberry, David Carrick, Caroline Haig, Nadeem Ahmed, Ify Mordi, Margaret McEntegart, Mark C. Petrie, Hany Eteiba, Stuart Hood, Stuart Watkins, Mitchell Lindsay, Andrew Davie, Ahmed Mahrous, Ian Ford, Naveed Sattar, Paul Welsh, Aleksandra Radjenovic, Keith G. Oldroyd, Colin Berry

**Affiliations:** a British Heart Foundation Glasgow Cardiovascular Research Centre, Institute of Cardiovascular and Medical Sciences, University of Glasgow, Glasgow, Scotland; b West of Scotland Heart and Lung Centre, Golden Jubilee National Hospital, Clydebank, Scotland; c Robertson Centre for Biostatistics, University of Glasgow, Glasgow, Scotland

**Keywords:** magnetic resonance imaging, myocardial infarction, remodeling, CMR, cardiac magnetic resonance, LV, left ventricular, MACE, major adverse cardiac event(s), STEMI, ST-segment elevation myocardial infarction

## Abstract

**Objectives:**

This study sought to determine the incidence and prognostic significance of persistent iron in patients post–ST-segment elevation myocardial infarction (STEMI).

**Background:**

The clinical significance of persistent iron within the infarct core after STEMI complicated by acute myocardial hemorrhage is poorly understood.

**Methods:**

Patients who sustained an acute STEMI were enrolled in a cohort study (BHF MR-MI [Detection and Significance of Heart Injury in ST Elevation Myocardial Infarction]). Cardiac magnetic resonance imaging including T_2_* (observed time constant for the decay of transverse magnetization seen with gradient-echo sequences) mapping was performed at 2 days and 6 months post-STEMI. Myocardial hemorrhage or iron was defined as a hypointense infarct core with T_2_* signal <20 ms.

**Results:**

A total of 203 patients (age 57 ± 11 years, n = 158 [78%] male) had evaluable T_2_* maps at 2 days and 6 months post-STEMI; 74 (36%) patients had myocardial hemorrhage at baseline, and 44 (59%) of these patients had persistent iron at 6 months. Clinical associates of persistent iron included heart rate (p = 0.009), the absence of a history of hypertension (p = 0.017), and infarct size (p = 0.028). The presence of persistent iron was associated with worsening left ventricular (LV) end-diastolic volume (regression coefficient: 21.10; 95% confidence interval [CI]: 10.92 to 31.27; p < 0.001) and worsening LV ejection fraction (regression coefficient: −6.47; 95% CI: −9.22 to −3.72; p < 0.001). Persistent iron was associated with the subsequent occurrence of all-cause death or heart failure (hazard ratio: 3.91; 95% CI: 1.37 to 11.14; p = 0.011) and major adverse cardiac events (hazard ratio: 3.24; 95% CI: 1.09 to 9.64; p = 0.035) (median follow-up duration 1,457 days [range 233 to 1,734 days]).

**Conclusions:**

Persistent iron at 6 months post-STEMI is associated with worse LV and longer-term health outcomes. (Detection and Significance of Heart Injury in ST Elevation Myocardial Infarction [BHF MR-MI]; NCT02072850)

## Graphical abstract

**Figure:**
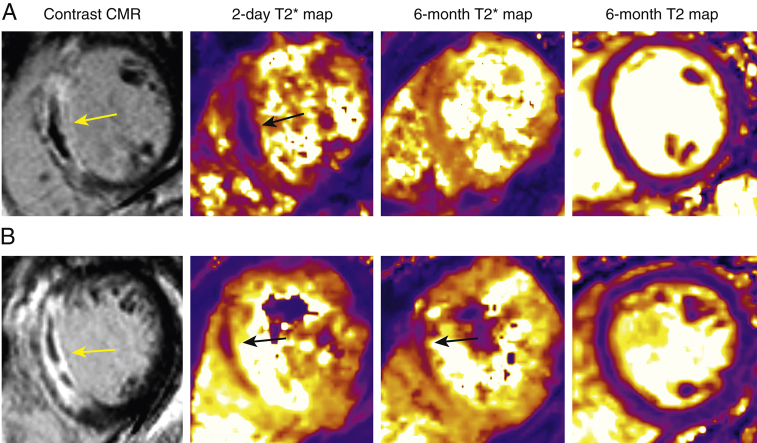

Myocardial hemorrhage (1) and microvascular obstruction (2) are common and prognostically important complications of reperfused ST-segment elevation myocardial infarction (STEMI), and they are independently associated with adverse remodeling and heart failure in the longer term (2). The improvements in survival after acute STEMI in recent decades translate to more surviving patients with injured hearts who are at risk of developing longer-term complications 3, 4. Because there are no evidence-based treatments for microvascular obstruction and myocardial hemorrhage, more research is needed to understand the pathophysiology of these disorders more fully.

Myocardial hemorrhage is a result of severe microvascular injury, with extravasation of erythrocytes secondary to loss of endothelial integrity 1, 5, 6, 7, 8. Hemoglobin degradation products are toxic 9, 10, 11, and their persistence is evidenced by immunohistochemical staining of iron within macrophages reflecting sustained inflammation within the infarct zone (10). Information relating to the clinical significance of persistent iron within the infarct core in patients with acute STEMI complicated by myocardial hemorrhage has been limited (e.g., sample size of n ≤ 40 11, 12, 13), and prognostic data on health outcomes are lacking.

We aimed to determine the incidence of persistent iron in a large cohort of STEMI survivors using contemporary T_2_* (observed time constant for the decay of transverse magnetization seen with gradient-echo sequences) mapping 14, 15. Additionally, we aimed to identify which clinical characteristics would be associated with persistent iron and whether persistent iron may be associated with adverse clinical outcomes.

We hypothesized that persisting iron would: 1) be associated with markers of the initial severity of STEMI; 2) present with distinct clinical characteristics when compared with resolved iron; 3) be associated with adverse myocardial remodeling; and 4) be associated with a worse prognosis in the longer term.

## Methods

The full methodology has been reported previously 16, 17, 18, 19 and is detailed in the Online Methods.

### Cardiac magnetic resonance image analysis

Cardiac magnetic resonance (CMR) imaging analysis was performed on a Siemens workstation (Siemen Healthcare, Erlangen, Germany). Left ventricular (LV) volumes and ejection fraction were assessed using computer-assisted planimetry (syngo.MR, Siemens Healthcare).

#### T_2_* measurement and myocardial hemorrhage

LV contours were delineated with computer-assisted planimetry on the raw T_2_* image and then copied onto color-coded spatially co-registered maps (Online Methods). Regions of interest were drawn in the infarct area surrounding core, core, and remote zones. Myocardial hemorrhage at 2 days and iron at 6 months were defined as regions of signal intensity <20 ms within the infarcted area and were measured as a percentage of LV mass and as a percentage of infarct size 20, 21, 22. Each T_2_* map was assessed by 2 independent CMR analysts for the presence of myocardial hemorrhage or iron.

#### T_2_ measurement and myocardial edema

LV contours on the last corresponding T_2_ (the transverse relaxation time)-weighted raw image with an echo time of 55 ms were planimetered and then copied to the map (23). Regions of interest were drawn in the surrounding infarct and remote zones. The extent of myocardial edema was defined as LV myocardium with pixel values (T_2_) >2 SD from remote myocardium 23, 24.

#### Infarct definition and size

The territory of infarction was quantified using computer-assisted planimetry and was expressed as a percentage of LV mass (25).

#### Myocardial salvage

Myocardial salvage was calculated by subtraction of percentage of infarct size from percentage of myocardial edema 7, 26, 27. The myocardial salvage index was calculated by dividing the myocardial salvage area by the initial percentage of myocardial edema.

#### Adverse remodeling

Adverse remodeling was defined as an increase in LV end-diastolic volume at 6 months from baseline by 20% or more (17).

### Health outcomes

We pre-specified adverse health outcomes that are implicated in the pathophysiology and natural history of STEMI. The primary composite outcome was all-cause death or first heart failure event (hospitalization for heart failure or defibrillator implantation) following the 6-month CMR scan. The secondary composite outcome was major adverse cardiac events (MACE).

### Statistical analysis

The full statistical methods are reported in the Online Methods. All p values were 2-sided. A p value >0.050 indicated the absence of a statistically significant effect. Analyses were performed using SPSS version 22 for Windows (SPSS, Inc., Chicago, Illinois), or R version 3.3.0 (R Foundation for Statistical Computing, Vienna, Austria).

## Results

Of 343 patients with STEMI referred for emergency percutaneous coronary intervention, 300 underwent serial CMR, 2.2 ± 1.9 days and 6 months after hospital admission. A total of 203 patients were included in the final analysis. The flow diagram for the study is shown in Online Figure 1. Please also refer to the Online Results.

### Patients’ characteristics

The characteristics of patients with paired evaluable T_2_* data (n = 203) are shown in Table 1 and Online Table 1. The mean ± SD age was 57 ± 11 years, and 78% were male.

**Table 1:** Characteristics of 203 Patients With Serial T_2_* Mapping 2 Days and 6 Months Post-STEMI, Grouped According to the Presence of Hemorrhage at 2 Days and the Persistence or Absence of Iron Within the Infarct Zone at 6 Months∗

	All Patients (N = 203)	No Acute Myocardial Hemorrhage (n = 129, 64%)	Acute Myocardial Hemorrhage 6 Months		p Value† R vs. P
			Resolved (R) (n = 30, 41%)‡	Persisting (P) (n = 44, 59%)‡	
Age, yrs	57 ± 11	58 ± 11	56 ± 12	57 ± 12	0.619
Male	158 (78)	93 (72)	25 (83)	40 (91)	0.471
Hypertension	61 (30)	37 (29)	14 (47)	10 (23)	0.043
Presenting characteristics					
Heart rate, beats/min	78 ± 16	77 ± 16	72 ± 14	85 ± 16	0.001
Culprit artery					
Left anterior descending	81 (40)	45 (35)	7 (23)	29 (66)	
Left circumflex	35 (17)	18 (14)	10 (33)	7 (16)	0.001
Right coronary	87 (43)	66 (51)	13 (43)	8 (18)	
Symptom onset to reperfusion, min	175 (122, 327)	170 (122, 310)	177 (129, 381)	208 (114, 402)	0.458
Reperfusion strategy					
Primary PCI	191 (94)	124 (96)	27 (90)	40 (91)	
Rescue PCI (failed thrombolysis)	8 (4)	2 (2)	2 (7)	4 (9)	0.637
Successful thrombolysis	4 (2)	3 (2)	1 (3)	0 (0)	
Blood results on admission					
Troponin I, ng/l	2,224 (684, 5,677) 1–28,406	1,567 (528, 2,784) 1–16,609	3,644 (439, 6,516) 3–8,561	6,531 (2,774, 10,330) 55–28,406	0.028

Values are mean ± SD, n (%), or median (Q1, Q3).

PCI = percutaneous coronary intervention; STEMI = ST-segment elevation myocardial infarction.

∗ Age, sex, and variables that differ between the groups are reported. The full table is reported in Online Table 1.

† The p values were obtained from Student’s *t* test, Fisher exact test or Mann-Whitney *U* test for comparisons between groups with resolved and persistent iron.

‡ Percentage of patients with hemorrhage at 2 days (n = 74).

A total of 74 (36%) patients had acute myocardial hemorrhage, and 44 (59%) of these patients had evidence of persistent iron at 6 months. No patients had de novo myocardial hemorrhage between the 2-day and 6-month scans.

Compared with patients with resolved hemorrhage from baseline, patients with persistent iron were less likely to have a history of hypertension, and they had higher heart rates at presentation (Table 1). The culprit artery was more likely to be the left anterior descending coronary artery, and these patients had higher peak troponin levels post-STEMI (Table 1).

### CMR findings

CMR findings were ascertained during the index hospitalization and at 6 months. The CMR findings are summarized in Table 2 and Online Table 2.

**Table 2:** CMR Findings at Baseline and at 6 Months in 203 Patients With STEMI Grouped According to the Presence of Hemorrhage at 2 Days and the Persistence or Absence of Iron Within the Infarct Zone at 6 Months∗

	All Patients (N = 203)	No Acute Myocardial Hemorrhage (n = 129, 64%)	Acute Myocardial Hemorrhage 6 Months		p Value† R vs. P
			Resolved (R) (n = 30, 49%)‡	Persistent (P) (n = 44, 59%)‡	
CMR findings 2 days post-MI (n = 211)					
LV ejection fraction, %	55 ± 10	57 ± 8	54 ± 9	47 ± 10	0.004
LV end-systolic volume, ml					
Men	76 ± 27	68 ± 23	78 ± 18	94 ± 31	0.012
Women	54 ± 14	52 ± 13	55 ± 16	71 ± 9	0.117
Edema and infarct characteristics at 2 days					
Myocardial edema, % LV mass	32 ± 12	29 ± 11	32 ± 10	42 ± 11	<0.001
Infarct size, % LV mass	18 ± 14	12 ± 10	22 ± 10	33 ± 12	<0.001
Late microvascular obstruction present	102 (50)	30 (23)	30 (100)	44 (100)	—
Late microvascular obstruction, % LV mass	2.5 ± 4.4	0.5 ± 1.6	4.1 ± 2.7	7.3 ± 6.4	0.005
Myocardial hemorrhage, % LV mass	8.5 ± 6.1	—	5.8 ± 4.0	10.3 ± 6.6	0.001
Myocardial hemorrhage, % infarct size	26.9 ± 15.2	—	27.2 ± 18.4	26.2 ± 12.8	0.684
CMR findings 6 months post-MI (n = 211)					
LV ejection fraction at 6 months, %	62 ± 10	65 ± 7	60 ± 7	53 ± 11	0.001
LV end-systolic volume at 6 months, ml					
Men	68 ± 36	55 ± 21	70 ± 21	98 ± 53	0.005
Women	48 ± 17	43 ± 15	55 ± 11	75 ± 14	0.045
Infarct characteristics at 6 months					
Infarct size, % LV mass	13 ± 10	9 ± 8	16 ± 7	24 ± 10	<0.001
Myocardial iron, % LV mass	2.4 ± 2.2	—	—	2.4 ± 2.2	—
Myocardial iron, % infarct size	10.6 ± 9.4	—	—	10.6 ± 9.4	—
Myocardial T_2_* values at 6 months					
T_2_* infarct at 6 months, ms	25.7 ± 4.4	27.1 ± 4.0	27.0 ± 4.3	21.6 ± 2.7	<0.001
T_2_* core at 6 months, ms	16.6 ± 2.1	—	—	16.6 ± 2.1	—

Values are mean ± SD or n (%).

CMR = cardiac magnetic resonance; LV = left ventricle; MI = myocardial infarction; STEMI = ST-segment elevation myocardial infarction.

∗ Extent of myocardial hemorrhage and variables that differ between the groups are reported. The full table is reported in Online Table 2.

† The p values were obtained from Student’s *t* test or Fisher exact test for comparisons between groups with resolved and persistent iron.

‡ Percentage of patients with hemorrhage at 2 days (n = 74).

#### CMR findings during the index hospitalization

The mean size of hemorrhage at baseline was 26.9 ± 15.2% of infarct size. All patients with acute myocardial hemorrhage had microvascular obstruction.

At 2-day CMR, patients with persisting iron had lower LV ejection fractions, larger LV end-systolic volumes, larger infarctions, a greater burden of microvascular obstruction, and a larger area of myocardial edema at baseline, compared with patients with resolved iron (Table 2). There was no difference in T_2_ signal in the infarct zone at baseline (Table 2).

#### CMR findings at 6 months

In patients with persistent iron, the extent of hemorrhage or iron (percentage of infarct size) reduced in size from baseline to follow-up (26.2 ± 12.8% vs. 10.6 ± 9.4%; p < 0.001) (Table 2).

**Figure 1:**
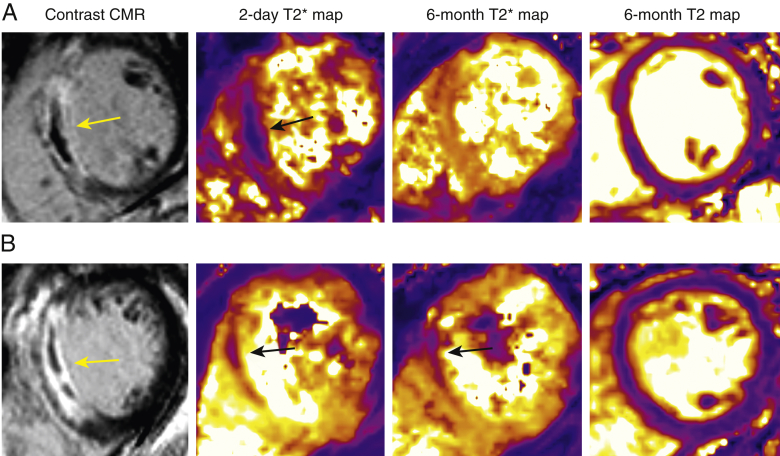
Two Patients With a Similar Presentation of Acute STEMI The full details are outlined in the Online Appendix. Contrast-enhanced cardiac magnetic resonance 2 days post-STEMI showed anteroseptal infarct in both patients **(left, yellow arrows)**. **(A)** Patient with resolved myocardial hemorrhage: T_2_* mapping at 2 days showed myocardial hemorrhage **(middle left, black arrow)** with resolution at 6 months **(middle right)**. The T_2_ value in the surrounding infarct region was 53 ms **(right)**. Left ventricular end-diastolic volume was unchanged from 126 to 127 ml in 6 months. This patient had an uncomplicated clinical course. **(B)** Patient with persisting iron: T_2_* mapping at 2 days showed myocardial hemorrhage **(middle left, black arrow)** that persisted at 6 months **(middle right, black arrow)**. The T_2_ value in the surrounding infarct region was 55 ms **(right)**. Left ventricular end-diastolic volume increased from 191 to 228 ml in 6 months. This patient was rehospitalized with new-onset heart failure. STEMI = ST-segment elevation myocardial infarction.

At 6 months, in patients with persistent iron, LV ejection fraction was lower and LV volumes and infarct size were higher compared with patients with resolved iron (Table 2). T_2_* values within the infarct zone were lower at 6 months in patients with persisting iron (Table 2, Online Figure 2). Compared with patients without hemorrhage at baseline, patients with hemorrhage at baseline had higher T_2_ values within the infarct zone at 6 months (58.7 ± 4.9 ms vs. 55.9 ± 3.7 ms; p < 0.001). Additionally, patients with persisting iron had higher infarct zone T_2_ values than patients without acute hemorrhage and patients with resolved iron collectively (59.5 ± 5.5 ms vs. 56.2 ± 3.8 ms; p = 0.001). There was no difference in T_2_ values within the infarct zone in patients with persisting iron compared with patients with resolved iron (Figures 1A and 1B, Online Table 2, Online Figure 2).

### Clinical associates of persistent iron

The multivariable associates of infarct core iron status at 6 months are shown in Table 3 and Online Table 3. The main predictors of persisting iron in patients with acute hemorrhage were a higher heart rate at presentation, the absence of a history of hypertension, and infarct size (Table 3).

**Table 3:** Multivariable Associations With 6-Month Iron Status (Resolved or Persisting) (n = 74) at 6 Months Post-STEMI in Logistic Regression Analysis∗

Multivariable Associations	Odds Ratio (95% CI)†	p Value
Patients’ characteristics and angiographic data		
Heart rate, beats/min	1.08 (1.02–1.14)	0.009
Hypertension	0.12 (0.02–0.68)	0.017
Patients’ characteristics, angiographic data, and infarct size‡		
Heart rate, beats/min	1.08 (1.01–1.16)	0.020
Hypertension	0.10 (0.01–0.67)	0.018
Infarct size, % LV mass	1.10 (1.01–1.20)	0.028

CI = confidence interval; other abbreviations as in Tables 1 and 2.

∗ Only statistically significant variables are reported. All variables included in the model are described in the Online Appendix.

† The odds ratio (95% CIs) indicates odds of persisting iron at 6 months given exposure to the independent variable.

‡ Similar results were obtained when myocardial edema was included instead of infarct size.

### Persistent iron and LV remodeling

In multivariable linear regression, persistent iron at 6 months was associated with worsening LV end-diastolic volume and worsening LV ejection fraction (Online Table 4, Online Figure 3). The multivariable association between persistent iron and adverse remodeling (odds ratio: 2.89; 95% confidence interval: 0.80 to 10.48; p = 0.106) was not statistically significant.

### Persistent iron and health outcome

**Figure 2:**
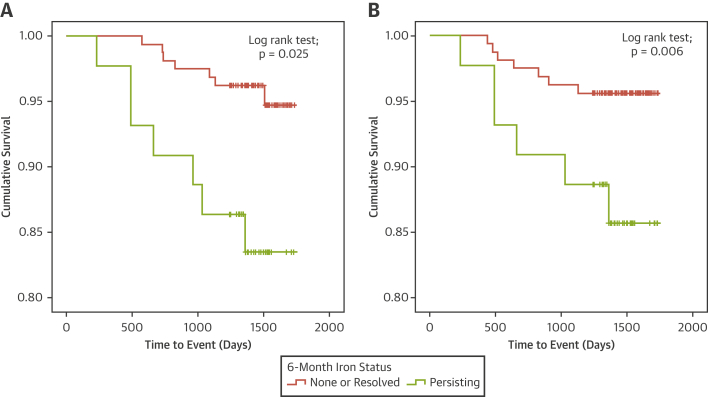
Persistent Iron and Adverse Outcomes After STEMI Kaplan-Meier survival curve for the relationship between infarct core iron status at 6 months and **(A)** all-cause death or heart failure and **(B)** major adverse cardiac events (censor time 1,457 days [range 233 to 1,734 days]). Persisting iron at 6 months post-ST-segment elevation myocardial infarction (STEMI) was associated with all-cause death or heart failure and major adverse cardiac events.

Health outcome data were available in 203 (100%) patients. The median duration of follow-up was 1,457 days (post-discharge censor duration range 233 to 1,734 days). All-cause death or heart failure following the 6-month assessment occurred in 14 (7%) patients, including 4 noncardiovascular deaths, 4 cardiovascular deaths (n = 2 sudden deaths), 1 undetermined cause of death, and 5 heart failure episodes (hospitalization for heart failure [n = 1] and defibrillator implantation [n = 4]). Persistent iron was associated with the occurrence of all-cause death or heart failure (hazard ratio: 3.91; 95% confidence interval: 1.37 to 11.14; p = 0.011) (Figure 2).

MACE following the 6-month assessment occurred in 13 (6%) patients, including 3 cardiovascular deaths (2 sudden deaths), 5 heart failure episodes (hospitalization for heart failure [n = 1] and defibrillator implantation [n = 4]), 4 non-STEMIs, and 1 STEMI.

Persistent iron was associated with the occurrence of MACE (hazard ratio: 3.24; 95% confidence interval: 1.09 to 9.64; p = 0.035) (Figure 2).

Associations with persistent iron and health outcome were not independent of the initial size of the infarct.

## Discussion

We present a large investigation of persistent iron within the infarct core, as revealed by T_2_* mapping, after acute myocardial hemorrhage in a cohort of unselected patients with STEMI.

The main findings are as follows: 1) 36% patients had myocardial hemorrhage at baseline, and 59% of these patients had evidence of persistent iron at 6 months; 2) de novo myocardial hemorrhage did not occur after the 2-day CMR scan; 3) clinical associates of persistent iron included patients’ characteristics (male sex, smoking status), hemodynamic features at presentation (heart rate), neutrophil count, and electrocardiographic, angiographic and imaging measures of STEMI severity (ST-segment resolution, Thrombus In Myocardial Infarction flow, infarct size, myocardial edema); 4) higher heart rate, absence of hypertension, and larger initial infarct size differentiated patients who had persisting iron from patients with resolution of iron; 5) persisting iron was associated with increasing LV end-diastolic volume and decreasing LV ejection fraction at 6 months; and 6) persisting iron was associated with an approximately 4-fold increase in the likelihood of all-cause death or heart failure and a 3-fold increase in the likelihood of MACE. Taken together, these findings identify persistent iron residues as a mechanistic explanation of LV remodeling and worsening function (Figures 1A and 1B). Potentially, persistent iron represents a therapeutic target, and further research seems warranted.

Our analysis builds on the results of other studies 11, 12, 18, and it helps to clarify some conflicting results (13). In a time-course study of myocardial edema and hemorrhage by Zia et al. (13), the mean T_2_* relaxation time returned to normal by 6 months post-STEMI, a finding implying that persistent iron is rare, whereas more recent studies indicated that persistent iron may be much more common 11, 12, 18. We think these differences can be explained by the emerging availability of T_2_* mapping methods, which have improved sensitivity and image quality.

Our results reveal that a history of hypertension may have a protective effect on the persistence of iron. In addition, a diagnosis of hypertension was associated with increasing LV ejection fraction. This is an unexpected finding, given that previous studies showed that hypertension is associated with myocardial hemorrhage acutely 28, 29. A history of hypertension reflects an established diagnosis and the presence of concomitant antihypertensive drug therapy initiated before the STEMI event. Further, persisting iron and acute myocardial hemorrhage reflect different but related processes. Persisting iron at 6 months reflects all factors from after reperfusion to follow-up, whereas myocardial hemorrhage early post-STEMI is related to acute reperfusion injury. We also observed no association between the time from symptom onset to reperfusion and the persistence of iron. Evidence suggests that ischemic time is associated with myocardial hemorrhage 6, 28, 30; however, studies in the present cohort 18, 19 and others 5, 11 have suggested that there is no association. Our results add to our idea that acute myocardial hemorrhage and persisting iron result from distinct pathological processes.

Bulluck et al. (12) pooled the results from all current studies of residual iron 11, 12, 18 and calculated the prevalence of myocardial hemorrhage as 39 of 73 (53%) patients, with 28 of 39 (72%) patients having persisting iron. Adding in our results (which include 30 patients from the serial imaging substudy by Carrick et al. [18]), the up-to-date values are 100 of 246 (41%) patients with myocardial hemorrhage (Kali et al. [11], 11 of 15; Bulluck et al. [12], 15 of 28; our study, 74 of 203) and 68 of 100 (68%) with persisting iron (Kali et al. [11], 11 of 11; Bulluck et al. [12], 13 of 15; our study, 44 of 74). The comparatively low incidence of persisting iron in our study may be a reflection of an unselected, consecutively recruited, large cohort of patients with STEMI, with a wide heterogeneity in the severity of infarcts. For example, in the population studied by Bulluck et al. (12), the acute infarct size was larger than in our study (27 ± 15% vs. 18 ± 14%), and the left anterior descending coronary culprit artery was predominant (60% vs. 40%). We have found that both these features are associated with persistence of iron residues.

Bulluck et al. (12) reported high T_2_ infarct zone signal in patients with persisting iron; however, the number of patients with resolved iron in their cohort was small (n = 2). Further, none of the patients in the study by Kali et al. (11) had resolved iron. The conclusion, therefore, that the persistence of iron causes edema has not been resolved. In the present study, myocardial T_2_ in the infarct zone at 6 months was higher in patients with acute myocardial hemorrhage, but no differences were observed in those patients with persistent iron compared with those with resolution (Figures 1A and 1B, Online Table 2). Other factors may be relevant, including the confounding problem that STEMI severity is linked with myocardial hemorrhage. Nonetheless, patients with persisting iron had higher infarct zone T_2_ signal than patients without hemorrhage and those with resolved iron collectively, a finding that supports a mechanistic basis for the association between persisting iron and worsening LV volumes and function. Persistent iron may represent a nidus to drive local and systemic inflammation, consistent with our observation of higher neutrophil counts in patients with persisting iron. This theory is further supported by a recent canine study by Kali et al. (10), which demonstrated the presence of proinflammatory cells in areas of iron deposition post-myocardial infarction.

Our research has important clinical implications. The persistence of iron defines a high-risk group of patients post-STEMI. Intramyocardial hemorrhage is proarrhythmic 31, 32, 33, and this feature may contribute in part to a higher mortality rate in patients with persisting iron at 6 months. The relationship between persistent iron and worsening health outcome further highlights the need for therapeutic interventions to prevent the occurrence of myocardial hemorrhage acutely. We have shown that patients with a more severe STEMI initially are at higher risk of persistent iron; therefore, novel treatments may be stratified to at-risk patients very early after reperfusion. Our results also support the case for CMR-based risk assessment at 6 months in those patients with acute myocardial hemorrhage early post-myocardial infarction to detect persistent infarct zone iron. Affected patients may benefit from more intensive therapy. We are uncertain about the justification for systemic iron chelation therapy as suggested by Bulluck et al. (34), given that iron deficiency is an adverse prognostic factor in patients with LV dysfunction (35). The possibility that patients with acute STEMI could benefit from targeted therapy to prevent myocardial hemorrhage is currently being investigated. T-TIME (A Trial of Low-dose Adjunctive alTeplase During prIMary PCI) (36) is a randomized, double-blind, placebo-controlled phase II trial of low-dose intracoronary alteplase in patients with acute STEMI who present <6 h from symptom onset with risk factors for microvascular obstruction (e.g., proximal culprit lesion location). T-TIME tests the efficacy hypothesis that intracoronary thrombolysis will reduce coronary thrombus burden, restore microvascular perfusion, reduce infarct zone hemorrhage, and improve surrogate clinical outcomes. The alternate safety hypothesis that intracoronary lysis will increase infarct zone hemorrhage and persistent myocardial iron, and thereby have an adverse effect on surrogate outcomes, will also be assessed.

### Study limitations

Our study lacks pathological correlation of the imaging results. Further, our results do not permit mechanistic interpretation regarding whether inflammation is the primary driver of persistent iron, or alternatively, persistent iron may reflect a defect in macrophage-mediated clearance of hemoglobin degradation products. As a result of time constraints imposed on the CMR examination, the T_2_* imaging protocol involved 3 short-axis slices (base, mid, apical) rather than a full LV stack, and therefore minor degrees of hemorrhage could have been missed. However, imaging positions were prescribed on anatomic landmarks, and scans were undertaken in the same laboratory, thus improving our ability to select the same matched slice positions between scans. The T_2_* acquisition was associated with imaging artifacts that limited the quantification of hemorrhage and iron in some patients. Future improvements to T_2_* mapping could include the use of high-pass filtered processing (37) and the use of an automated truncation method (38). Because the survival analyses included 14 events, we were limited in the number of confounders we could account for in the statistical models. These results are preliminary, and further research is warranted.

## Conclusions

Persistent iron within the infarct core is common (about 3 in 5) in patients with myocardial hemorrhage early post-STEMI. Persistent iron is predictive of worsening LV function and volumes, as well as all-cause death or heart failure and MACE in the longer term.

Perspectives **COMPETENCY IN MEDICAL KNOWLEDGE:** Myocardial hemorrhage that occurs acutely after STEMI can persist as infarct core iron in the long term in approximately 3 in 5 patients. Persistent iron is predictive of worsening LV function and volumes, all-cause death or heart failure, and MACE in the longer term.

**COMPETENCY IN PATIENT CARE AND PROCEDURAL SKILLS:** The persistence of myocardial iron can be predicted on the basis of the initial severity of the myocardial infarction. In patients with acute myocardial hemorrhage, repeat CMR at 6 months may be useful for risk stratification. Patients who present with more severe infarcts may be targeted with novel treatments, such as intracoronary thrombolysis. Further investigation is warranted.

**TRANSLATIONAL OUTLOOK:** Survival analysis was limited by the small number of events (n = 14). Therefore, the results are hypothesis generating.

## References

[1] Higginson L.A., White F., Heggtveit H.A., Sanders T.M., Bloor C.M., Covell J.W. (1982). Determinants of myocardial hemorrhage after coronary reperfusion in the anesthetized dog. Circulation.

[2] van Kranenburg M., Magro M., Thiele H. (2014). Prognostic value of microvascular obstruction and infarct size, as measured by CMR in STEMI patients. J Am Coll Cardiol Img.

[3] Moran A.E., Forouzanfar M.H., Roth G.A. (2014). The global burden of ischemic heart disease in 1990 and 2010: the Global Burden of Diseases 2010 study. Circulation.

[4] Chen J., Hsieh A.F., Dharmarajan K., Masoudi F.A., Krumholz H.M. (2013). National trends in heart failure hospitalization after acute myocardial infarction for Medicare beneficiaries: 1998-2010. Circulation.

[5] Ganame J., Messalli G., Dymarkowski S. (2009). Impact of myocardial haemorrhage on left ventricular function and remodelling in patients with reperfused acute myocardial infarction. Eur Heart J.

[6] Amabile N., Jacquier A., Shuhab A. (2012). Incidence, predictors, and prognostic value of intramyocardial hemorrhage lesions in ST elevation myocardial infarction. Catheter Cardiovasc Interv.

[7] Eitel I., Kubusch K., Strohm O. (2011). Prognostic value and determinants of a hypointense infarct core in T2-weighted cardiac magnetic resonance in acute reperfused ST-elevation-myocardial infarction. Circ Cardiovasc Imaging.

[8] Husser O., Monmeneu J.V., Sanchis J. (2013). Cardiovascular magnetic resonance-derived intramyocardial hemorrhage after STEMI: influence on long-term prognosis, adverse left ventricular remodeling and relationship with microvascular obstruction. Int J Cardiol.

[9] Rifkind J.M., Mohanty J.G., Nagababu E. (2015). The pathophysiology of extracellular hemoglobin associated with enhanced oxidative reactions. Front Physiol.

[10] Kali A., Cokic I., Tang R. (2016). Persistent microvascular obstruction after myocardial infarction culminates in the confluence of ferric iron oxide crystals, proinflammatory burden, and adverse remodeling. Circ Cardiovasc Imaging.

[11] Kali A., Kumar A., Cokic I. (2013). Chronic manifestation of postreperfusion intramyocardial hemorrhage as regional iron deposition: a cardiovascular magnetic resonance study with ex vivo validation. Circ Cardiovasc Imaging.

[12] Bulluck H., Rosmini S., Abdel-Gadir A. (2016). Residual myocardial iron following intramyocardial hemorrhage during the convalescent phase of reperfused ST-segment-elevation myocardial infarction and adverse left ventricular remodeling. Circ Cardiovasc Imaging.

[13] Zia M.I., Ghugre N.R., Connelly K.A. (2012). Characterizing myocardial edema and hemorrhage using quantitative T2 and T2* mapping at multiple time intervals post ST-segment elevation myocardial infarction. Circ Cardiovasc Imaging.

[14] Kali A., Tang R.L., Kumar A., Min J.K., Dharmakumar R. (2013). Detection of acute reperfusion myocardial hemorrhage with cardiac MR imaging: T2 versus T2*. Radiology.

[15] Kumar A., Green J.D., Sykes J.M. (2011). Detection and quantification of myocardial reperfusion hemorrhage using T2*-weighted CMR. J Am Coll Cardiol Img.

[16] Detection and Significance of Heart Injury in ST Elevation Myocardial Infarction. Available at: https://clinicaltrials.gov/ct2/show/NCT02072850. Accessed July 31, 2016.

[17] Carrick D., Haig C., Rauhalammi S. (2015). Pathophysiology of LV Remodeling in survivors of STEMI: inflammation, remote myocardium, and prognosis. J Am Coll Cardiol Img.

[18] Carrick D., Haig C., Ahmed N. (2016). Temporal evolution of myocardial hemorrhage and edema in patients after acute ST-segment elevation myocardial infarction: pathophysiological insights and clinical implications. J Am Heart Assoc.

[19] Carrick D., Haig C., Ahmed N. (2016). Myocardial hemorrhage after acute reperfused ST-segment-elevation myocardial infarction relation to microvascular obstruction and prognostic significance. Circ Cardiovasc Imaging.

[20] Kandler D., Lücke C., Grothoff M. (2014). The relation between hypointense core, microvascular obstruction and intramyocardial haemorrhage in acute reperfused myocardial infarction assessed by cardiac magnetic resonance imaging. Eur Radiol.

[21] O’Regan D.P., Ariff B., Neuwirth C., Tan Y., Durighel G., Cook S.A. (2010). Assessment of severe reperfusion injury with T2* cardiac MRI in patients with acute myocardial infarction. Heart.

[22] Ghugre N.R., Ramanan V., Pop M. (2011). Quantitative tracking of edema, hemorrhage, and microvascular obstruction in subacute myocardial infarction in a porcine model by MRI. Magn Reson Med.

[23] Wassmuth R., Prothmann M., Utz W. (2013). Variability and homogeneity of cardiovascular magnetic resonance myocardial T2-mapping in volunteers compared to patients with edema. J Cardiovasc Magn Reson.

[24] Payne A.R., Casey M., McClure J. (2011). Bright-blood T2-weighted MRI has higher diagnostic accuracy than dark-blood short tau inversion recovery MRI for detection of acute myocardial infarction and for assessment of the ischemic area at risk and myocardial salvage. Circ Cardiovasc Imaging.

[25] Flett A.S., Hasleton J., Cook C. (2011). Evaluation of techniques for the quantification of myocardial scar of differing etiology using cardiac magnetic resonance. J Am Coll Cardiol Img.

[26] Francone M., Bucciarelli-Ducci C., Carbone I. (2009). Impact of primary coronary angioplasty delay on myocardial salvage, infarct size, and microvascular damage in patients with ST-segment elevation myocardial infarction: insight from cardiovascular magnetic resonance. J Am Coll Cardiol.

[27] Payne A.R., Berry C., Doolin O. (2012). Microvascular resistance predicts myocardial salvage and infarct characteristics in ST-elevation myocardial infarction. J Am Heart Assoc.

[28] Garcia-Dorado D., Théroux P., Solares J. (1990). Determinants of hemorrhagic infarcts: histologic observations from experiments involving coronary occlusion, coronary reperfusion, and reocclusion. Am J Pathol.

[29] Verma S., Fedak P.W., Weisel R.D. (2002). Fundamentals of reperfusion injury for the clinical cardiologist. Circulation.

[30] Kloner R.A., Rude R.E., Carlson N., Maroko P.R., DeBoer L.W., Braunwald E. (1980). Ultrastructural evidence of microvascular damage and myocardial cell injury after coronary artery occlusion: which comes first?. Circulation.

[31] Mather A.N., Fairbairn T.A., Ball S.G., Greenwood J.P., Plein S. (2011). Reperfusion haemorrhage as determined by cardiovascular MRI is a predictor of adverse left ventricular remodelling and markers of late arrhythmic risk. Heart.

[32] Cokic I., Kali A., Yang H.J. (2015). Iron-sensitive cardiac magnetic resonance imaging for prediction of ventricular arrhythmia risk in patients with chronic myocardial infarction. Circ Cardiovasc Imaging.

[33] Cokic I., Kali A., Wang X. (2013). Iron deposition following chronic myocardial infarction as a substrate for cardiac electrical anomalies: initial findings in a canine model. PLoS One.

[34] Dharmakumar R. (2016). “Rusty hearts.”. Circ Cardiovasc Imaging.

[35] Jankowska E.A., Rozentryt P., Witkowska A. (2010). Iron deficiency: an ominous sign in patients with systolic chronic heart failure. Eur Heart J.

[36] A Trial of Low-dose Adjunctive alTeplase During prIMary PCI. Available at: https://clinicaltrials.gov/ct2/show/NCT02257294. Accessed March 27, 2017.

[37] Goldfarb J.W., Hasan U., Zhao W., Han J. (2014). Magnetic resonance susceptibility weighted phase imaging for the assessment of reperfusion intramyocardial hemorrhage. Magn Reson Med.

[38] Sandino C.M., Kellman P., Arai A.E., Hansen M.S., Xue H. (2015). Myocardial T2* mapping: influence of noise on accuracy and precision. J Cardiovasc Magn Reson.

