# Supplementary material for: Persistent Iron Within the Infarct Core After ST-Segment Elevation Myocardial Infarction: Implications for Left Ventricular Remodeling and Health Outcomes
Source: JACC Cardiovasc Imaging. 2018 Sep;11(9):1248–56. doi: 10.1016/j.jcmg.2017.08.027 (PMC6130225; doi:10.1016/j.jcmg.2017.08.027)
Supplement: Online Data [file mmc1.docx]

# SUPPLEMENTAL MATERIAL

# Persistent iron within the infarct core after ST-elevation myocardial infarction: implications for left ventricular remodeling and health outcomes.

# ClinicalTrials.gov registration NCT02072850Table of contents

Supplementary Methods 3

Supplementary Results 10

Supplementary Tables 11

Supplementary Figure Legends 25

Supplementary Figures 26

References 29

# Supplementary Methods

## Setting and study populations

### ST-elevation myocardial infarction patients

We performed a cardiac magnetic resonance (CMR) imaging longitudinal cohort study in a regional cardiac center between 11 May 2011 and 22 November 2012. Near-consecutive patients with acute ST-elevation myocardial infarction (STEMI) provided written informed consent. Inclusion criteria were an indication for primary percutaneous coronary intervention (PCI) or thrombolysis for STEMI (1). Exclusion criteria were contraindications to contrast-enhanced CMR. The study was approved by the National Research Ethics Service (Reference 10-S0703-28) and was publically registered (NCT02072850 (2)).

Screening, enrolment, and data collection were prospectively performed by cardiologists in the cardiac catheterization laboratories of the Golden Jubilee National Hospital, Glasgow, United Kingdom. This hospital is a regional referral center for primary and rescue PCI. The hospital provides clinical services for a population of 2.2 million. A screening log was recorded, including patients who did not participate in the cohort study.

## Coronary angiogram acquisition and analyses

Coronary angiograms were acquired during usual care with cardiac catheter laboratory X-ray (Innova®) and IT equipment (Centricity®) made by GE Healthcare.

## Percutaneous coronary intervention

Consecutive admissions with acute STEMI referred for emergency PCI were screened for the inclusion and exclusion criteria. During ambulance transfer to the hospital, the patients received 300 mg of aspirin, 600 mg of clopidogrel and 5000 IU of unfractionated heparin (1,3). The initial primary PCI procedure was performed using radial artery access. A conventional approach to primary PCI was adopted in line with usual care in our hospital (1,3). Conventional bare metal and drug eluting stents were used in line with guideline recommendations and clinical judgment. The standard transcatheter approach for reperfusion involves minimal intervention with aspiration thrombectomy only or minimal balloon angioplasty (e.g. a compliant balloon sized according to the reference vessel diameter and inflated at 4-6 atmospheres 1-2 times). During PCI, glycoprotein IIbIIIa inhibitor therapy was initiated with high dose tirofiban (25 μg/kg/bolus) followed by an intravenous infusion of 0.15 μg/kg/min for 12 hours, according to clinical judgment and indications for bail-out therapy (1,3). No reflow was treated according to contemporary standards of care with intra-coronary nitrate (i.e. 200 μg) and adenosine (i.e. 30 – 60 μg) (1,3), as clinically appropriate. In patients with multivessel coronary disease, multivessel PCI was not recommended, in line with clinical guidelines (1,3). The subsequent management of these patients was symptom-guided.

## Angiographic analysis

The coronary anatomy and disease characteristics of study participants were described based on the clinical reports of the attending cardiologist.

## Outcome definitions

Coronary blood flow can be described based on the visual assessment of coronary blood flow revealed by contrast injection into the coronary arteries (1,3). Thrombus in Myocardial Infarction (TIMI) Coronary Flow Grade 0 is no flow, 1 is minimal flow past obstruction, 2 is slow (but complete) filling and slow clearance, and 3 is normal flow and clearance.

## CMR acquisition

CMR was performed on a Siemens MAGNETOM Avanto (Erlangen, Germany) 1.5-Tesla scanner with a 12-element phased array cardiac surface coil 2 days and 6 months post-MI. The imaging protocol included cine CMR with steady-state free precession for imaging structure and function.

T1-mapping was performed pre- and 15 minutes post-gadolinium contrast administration. T1 maps were acquired in 3 short-axial slices (basal, mid and apical), using a modified look-locker inversion-recovery (MOLLI) investigational prototype sequence (Work-in-Progress (WIP) method 448, Siemens Healthcare) (4–6). The CMR parameters were: bandwidth ~1090 Hz/pixel; flip angle 35°; echo time (TE) 1.1 ms; T1 of first experiment 100 ms; TI increment 80 ms; matrix 192 x 124 pixels; spatial resolution 2.2 x 1.8 x 8.0 mm; slice thickness 8 mm; scan time 17 heartbeats.

T2* maps were obtained using an investigational prototype T2* map sequence acquired in 3 short-axis slices (basal, mid and apical). Typical imaging parameters were: bandwidth ~814 (x8) Hz/pixel; flip angle 18°; matrix 256x115; spatial resolution 2.6 x 1.6 x 10 mm; slice thickness 8 mm.

T2-mapping (WIP method 447, Siemens Healthcare) was acquired in contiguous short axis slices covering the whole left ventricle (LV), using an investigational prototype T2-prepared TrueFisp sequence (7,8). The CMR parameters were: bandwidth ~947 Hz/pixel; flip angle 70°; T2 preparations: 0 ms, 24 ms, and 55 ms respectively; matrix 160 x 105 pixels; spatial resolution 2.6 x 2.1 x 8.0 mm; slice thickness 8 mm.

Late gadolinium enhancement images covering the entire LV were acquired 10-15 minutes after intravenous injection of 0.15 mmol/kg of gadoterate meglumine (Gd2+-DOTA, Dotarem, Guebert S.A.) using segmented phase-sensitive inversion recovery turbo fast low-angle shot (9). Typical imaging parameters were: bandwidth ~130 Hz/pixel, flip angle 25°, TE 3.36 ms, matrix 192 x 256 pixels, echo spacing 8.7ms and trigger pulse 2. The voxel size was 1.8 x 1.3 x 8 mm3. Inversion times were individually adjusted to optimize nulling of apparently normal myocardium (typical values, 200 to 300 ms).

## CMR image analyses

### T2* measurement and myocardial hemorrhage

LV contours were delineated with computer-assisted planimetry on the raw T2* image. Care was taken to have adequate margins of separation from tissue interfaces prone to partial volume effects (10–12). Each T2* map image was assessed for the presence of artefacts relating to susceptibility effects or cardio-respiratory motion. Each map was evaluated against the original images. When artefacts occurred, the affected segments were not included in the analysis.

### Extracellular volume

LV contours were delineated on the best spatially matched raw T1 image and copied onto color-coded spatially co-registered maps. Regions of interest were drawn in infarcted myocardium, remote myocardium and LV blood pool. Extracellular volume (ECV) was calculated as a ratio of corresponding T1 values measured pre- and post- contrast in each of the regions of interest. ECV was calculated using ECV = (1-HCT) × λ, where Lambda (λ)=ΔR1myocardium/ΔR1blood, ΔR1=R1post-contrast-R1pre-contrast and R1=1/T1 (13,14). Hematocrit (HCT) was measured at the time of scanning.

### Reference ranges

Reference ranges used in the laboratory were 105 – 215 g for LV mass in men, 70 – 170 g for LV mass in women, 77 – 195 ml for LV end-diastolic volume in men, 52 – 141 ml for LV end-diastolic volume in women, 19 – 72 ml for LV end-systolic volume in men and 13 – 51 ml for LV end-systolic volume in women.

## Electrocardiogram

A 12 lead ECG was obtained before coronary reperfusion and 60 minutes afterwards with Mac-Lab® technology (GE Healthcare) in the catheter laboratory and a MAC 5500 HD recorder (GE Healthcare) in the Coronary Care Unit. The ECGs were acquired by trained cardiology staff. The ECGs were de-identified and transferred to the local ECG management system. The ECGs were then analyzed by the University of Glasgow ECG Core Laboratory which is certified to ISO 9001: 2008 standards as a UKAS Accredited Organization.

The extent of ST-segment resolution on the ECG assessed 60 minutes after reperfusion compared to the baseline ECG before reperfusion (1) was expressed as complete (≥70%), incomplete (>30% to < 70%) or none (≤30%).

## Biochemical measurement of infarct size

Troponin T was measured (Elecsys Troponin T, Roche) as a biochemical measure of infarct size. The high sensitive assay reaches a level of detection of 5 pg/ml and achieves less than 10% variation at 14 pg/ml corresponding to the 99th percentile of a reference population. A blood sample was routinely obtained 12 – 24 hours after hospital admission, and again between 0700 - 0900 hours during the first two days of the index hospitalization.

## Biochemical measurement of LV remodeling

Serial systemic blood sample were obtained immediately after reperfusion in the cardiac catheterization laboratory, and subsequently between 0600 - 0700 hrs each day during the initial in-patient stay in the Coronary Care Unit.

NT-proBNP, a biochemical measure of LV wall stress, was measured in a research laboratory using an electrochemiluminescence method (e411, Roche) and the manufacturers’ calibrators and quality control material. The limit of detection is 5 pg/ml. Long-term coefficient of variations of low and high controls are typically <5%, and were all within the manufacturers’ range.

## **Research management**

The study was conducted in line with Guidelines for Good Clinical Practice (GCP) in Clinical Trials (15).

Trial management included a Trial Management Group, and an independent Clinical Trials Unit. Day to day study activity was coordinated by the Trial Management Group who was responsible to the Sponsor which was responsible for overall governance and that the trial was conducted according to GCP standards.

## Health outcomes

We prespecified adverse health outcomes that are pathophysiologically linked with STEMI. The primary composite outcome was all-cause death or first heart failure event (hospitalization for heart failure or defibrillator implantation) following the 6 month CMR scan.

Research staff screened for events from enrollment by checking the medical records and by contacting patients and their primary and secondary care physicians as appropriate. Each serious adverse event was reviewed by a cardiologist who was independent of the research team and blinded to all of the clinical and CMR data. The serious adverse events were defined according to standard guidelines (16).

## Statistical analysis

Continuous variables are described as mean±SD, if normally distributed, and median (Q1, Q3) otherwise. Categorical variables are described as n (%). Patients were grouped by no hemorrhage at any time point, hemorrhage at baseline which resolves at follow-up, and hemorrhage which persists as infarct core iron at follow-up. Group characteristics and CMR findings were compared using independent sample t-tests, Mann Whitney tests or Fisher’s tests, as appropriate. Logistic regression was used to identify associates of infarct core iron status at 6 months. Multivariable linear regression analyses using the enter method were performed to identify associates of LV parameters. Linear regression assumptions were verified using standardized residual plots.

Cox proportional hazards regression was used to explore potential associations between 6-month infarct core iron status and health outcome. The proportional hazards assumption was verified using log-minus-log plots. For these plots, continuous variables were categorized as above and below the median.

# Supplementary Results

## Extracellular volume

In the subset of patients (n=155) with ECV available at 6 months, infarct zone ECV tended to be higher in patients with persistent iron. Remote zone ECV was higher in women with persistent iron (Supplementary Table 2).

## Associates with iron status at 6 months post-STEMI

The clinical characteristics that were included in the multivariable model with myocardial iron status at 6 months post-STEMI (resolved or persisting) were age (p=0.332), culprit artery percentage stenosis (p=0.190), symptom onset to reperfusion time (p=0.394), body mass index (BMI) (p=0.354), systolic blood pressure per 10 mmHg (p=0.133), diabetes mellitus (p=0.759), previous MI (p=0.893), previous angina (p=0.737), current smoker (p=0.412), TIMI flow grade 0/1/2 post-PCI vs. TIMI flow grade 3 post-PCI (reference category) (p=0.250), no ST-segment resolution vs. partial or complete ST-segment resolution (reference category) (p=0.175).

The multivariable predictors are described in Table 3 in the main paper.

# Supplementary Tables

**Supplementary Table 1.** Characteristics of 203 patients with serial T2* mapping 2 days and 6 months post-ST-elevation myocardial infarction, grouped according to the presence of hemorrhage at 2 days and the persistence or absence of iron within the infarct zone at 6 months.

| Characteristics |  | All patients | No acute myocardial hemorrhage | Acute myocardial hemorrhage | | P-value* |
| --- | --- | --- | --- | --- | --- | --- |
|  |  |  |  | 6 months | |  |
|  |  |  |  | Resolved (R) | Persisting (P) | R vs. P |
|  |  | n=203 | n=129 (64%) | n=30 (41%)† | n=44 (59%)† |  |
| Age, years |  | 57±11 | 58±11 | 56±12 | 57±12 | 0.619 |
| Male, n (%) |  | 158 (78) | 93 (72) | 25 (83) | 40 (91) | 0.471 |
| BMI, kg/m^2^ |  | 28±4 | 28±4 | 29±4 | 28±5 | 0.361 |
| Hypertension, n (%) |  | 61 (30) | 37 (29) | 14 (47) | 10 (23) | 0.043 |
| Current smoking, n (%) |  | 126 (62) | 73 (57) | 22 (73) | 31 (71) | 1.000 |
| Hypercholesterolemia, n (%) |  | 53 (26) | 30 (23) | 7 (23) | 16 (36) | 0.309 |
| Diabetes mellitus‡, n (%) |  | 25 (12) | 13 (10) | 6 (20) | 6 (13) | 0.530 |
| Previous angina, n (%) |  | 24 (12) | 14 (11) | 5 (17) | 5 (11) | 0.514 |
| Previous myocardial infarction, n (%) |  | 10 (5) | 6 (5) | 1 (3) | 3 (7) | 0.642 |
| Previous PCI, n (%) |  | 6 (3) | 3 (2) | 0 (0) | 3 (7) | 0.267 |
| *Presenting characteristics* |  |  |  |  |  |  |
| Heart rate, bpm |  | 78±16 | 77±16 | 72±14 | 85±16 | 0.001 |
| Systolic blood pressure, mmHg |  | 137±25 | 136±26 | 134±25 | 140±21 | 0.334 |
| Diastolic blood pressure, mmHg |  | 80±14 | 78±14 | 83±14 | 83±14 | 0.931 |
| Symptom onset to reperfusion, min | median (Q1, Q3)  range | 175 (122, 327)  68 - 1394 | 170 (122, 310)  68 - 1394 | 177 (129, 381)  88 - 705 | 208 (114, 402)  77 - 994 | 0.458 |
| Ventricular fibrillation§, n (%) |  | 10 (5) | 7 (5) | 1 (3) | 2 (5) | 1.000 |
| Killip class\|\|, n (%) | I | 147 (72) | 102 (79) | 22 (73) | 23 (52) |  |
|  | II | 43 (21) | 26 (20) | 6 (20) | 11 (25) | 0.122 |
|  | III/IV | 13 (6) | 1 (1) | 2 (7) | 10 (23) |  |
| *ECG* |  |  |  |  |  |  |
| ST-segment resolution post-PCI, n (%) | Complete, ≥70 % | 94 (47) | 70 (55) | 13 (43) | 11 (25) |  |
|  | Incomplete, 30% to <70% | 77 (38) | 44 (34) | 13 (43) | 20 (46) | 0.144 |
|  | None, ≤30% | 31 (15) | 14 (11) | 4 (13) | 13 (30) |  |
| *Coronary angiography* |  |  |  |  |  |  |
| Reperfusion strategy, n (%) | Primary PCI | 191 (94) | 124 (96) | 27 (90) | 40 (91) |  |
|  | Rescue PCI (failed thrombolysis) | 8 (4) | 2 (2) | 2 (7) | 4 (9) | 0.637 |
|  | Successful thrombolysis | 4 (2) | 3 (2) | 1 (3) | 0 (0) |  |
| Number of diseased arteries¶, n (%) | 1 | 114 (56) | 67 (52) | 18 (60) | 29 (67) |  |
|  | 2 | 59 (29) | 41 (32) | 6 (20) | 12 (27) | 0.314 |
|  | 3 | 25 (12) | 17 (13) | 5 (17) | 3 (7) |  |
|  | Left main | 5 (3) | 4 (3) | 1 (3) | 0 (0) |  |
| Culprit artery, n (%) | Left anterior descending | 81 (40) | 45 (35) | 7 (23) | 29 (66) |  |
|  | Left circumflex | 35 (17) | 18 (14) | 10 (33) | 7 (16) | 0.001 |
|  | Right coronary | 87 (43) | 66 (51) | 13 (43) | 8 (18) |  |
| Culprit artery TIMI flow grade at initial angiography, n (%) | 0/1 | 150 (74) | 82 (64) | 28 (93) | 40 (91) |  |
|  | 2 | 34 (17) | 29 (23) | 2 (7) | 3 (7) | 1.000 |
|  | 3 | 19 (9) | 18 (14) | 0 (0) | 1 (2) |  |
| Culprit artery TIMI flow grade post-PCI, n (%) | 0/1 | 2 (1) | 1 (1) | 0 (0) | 1 (2) |  |
|  | 2 | 7 (3) | 4 (3) | 1 (3) | 2 (5) | 1.000 |
|  | 3 | 194 (96) | 124 (96) | 29 (97) | 41 (93) |  |
| *Blood results on admission* |  |  |  |  |  |  |
| Troponin I, ng/L | median (Q1, Q3)  range | 2224 (684, 5677)  1 - 28406 | 1567 (528, 2784)  1 - 16609 | 3644 (439, 6516)  3 - 8561 | 6531 (2774, 10330)  55 - 28406 | 0.028 |
| C-reactive protein, mg/L | median (Q1, Q3)  range | 3 (2, 7)  1 - 125 | 4 (2, 7)  1 - 68 | 2 (2, 7)  1 - 24 | 3 (1, 6)  1 - 125 | 0.491 |
| NT-proBNP, pg/mL | median (Q1, Q3)  range | 729 (352, 1591)  87 - 19521 | 606 (301, 1415)  87 - 19521 | 1047 (564, 1629)  87 - 5301 | 846 (406, 1645)  198 - 5383 | 0.879 |
| Leucocytes, x10^9^L |  | 12.3±3.6 | 11.7±3.2 | 12.9±3.2 | 13.9±4.3 | 0.301 |
| Neutrophils, x10^9^L |  | 9.5±3.4 | 8.8±2.9 | 10.0±3.3 | 11.3±4.0 | 0.171 |
| Monocytes, x10^9^L |  | 0.8±0.3 | 0.8±0.3 | 0.9±0.4 | 0.9±0.4 | 0.811 |
| *Medical therapy* |  |  |  |  |  |  |
| Aspirin, n (%) |  | 202 (99) | 128 (99) | 30 (100) | 44 (100) | - |
| Clopidogrel, n (%) |  | 201 (99) | 127 (98) | 30 (100) | 44 (100) | - |
| Beta-blocker, n (%) |  | 198 (98) | 125 (97) | 29 (97) | 44 (100) | 0.405 |
| ACE-I or ARB, n (%) |  | 201 (99) | 127 (98) | 30 (100) | 44 (100) | - |
| Statin, n (%) |  | 203 (100) | 129 (100) | 30 (100) | 44 (100) | - |

Footnote: Abbreviations: ACE-I = angiotensin converting enzyme inhibitor, ARB = angiotensin receptor blocker, TIMI = Thrombus in Myocardial Infarction, PCI = percutaneous coronary intervention.

Data are given as n (%), mean±SD, or median (Q1, Q3) where appropriate. *P-values were obtained from t-test, Fisher’s test or Mann-Whitney test for comparisons between groups with resolved and persistent iron. †Percentage of patients with hemorrhage at 2 days (n=74).

‡History of diet-controlled or treated diabetes. §Successfully electrically cardioverted ventricular fibrillation at presentation or during PCI. ||Killip classification of heart failure post-MI: class I - no heart failure, class II - pulmonary rales or crepitations, third heart sound, and elevated jugular venous pressure, class III - acute pulmonary edema, class IV - cardiogenic shock. ¶Number of stenoses ≤50% of the reference vessel diameter by visual assessment and if there was left main stem involvement.

**Supplementary Table 2.** CMR findings at baseline and at 6 months in 203 ST-elevation myocardial infarction patients grouped according to the presence of hemorrhage at 2 days and the persistence or absence of iron within the infarct zone at 6 months.

| Characteristics | All patients | No acute myocardial hemorrhage | Acute myocardial hemorrhage | | P-value* |
| --- | --- | --- | --- | --- | --- |
|  |  |  | 6 months | |  |
|  |  |  | Resolved (R) | Persistent (P) | R vs. P |
|  | n=203 | n=129 (64%) | n=30 (49%)† | n=44 (59%)† |  |
| *CMR findings 2 days post-MI (n=211)* |  |  |  |  |  |
| LV ejection fraction, % | 55±10 | 57±8 | 54±9 | 47±10 | 0.004 |
| LV end-diastolic volume, ml |  |  |  |  |  |
| Men | 163±31 | 155±29 | 168±22 | 177±36 | 0.216 |
| Women | 124±21 | 123±22 | 133±26 | 121±6 | 0.380 |
| LV end-systolic volume, ml |  |  |  |  |  |
| Men | 76±27 | 68±23 | 78±18 | 94±31 | 0.012 |
| Women | 54±14 | 52±13 | 55±16 | 71±9 | 0.117 |
| LV mass, g |  |  |  |  |  |
| Men | 147±35 | 141±30 | 149±32 | 159±45 | 0.343 |
| Women | 95±21 | 92±19 | 112±28 | 106±8 | 0.684 |
| *Edema and infarct characteristics at baseline* |  |  |  |  |  |
| Myocardial edema, % LV mass | 32±12 | 29±11 | 32±10 | 42±11 | <0.001 |
| Infarct size, % LV mass | 18±14 | 12±10 | 22±10 | 33±12 | <0.001 |
| Myocardial salvage, % LV mass | 19±9 | 20±10 | 16±8 | 18±8 | 0.284 |
| Myocardial salvage index, % LV mass | 62±24 | 71±23 | 48±17 | 43±17 | 0.185 |
| Late microvascular obstruction present, n (%) | 102 (50) | 30 (23) | 30 (100) | 44 (100) | - |
| Late microvascular obstruction, % LV mass | 2.5±4.4 | 0.5±1.6 | 4.1±2.7 | 7.3±6.4 | 0.005 |
| Myocardial hemorrhage, % LV mass | 8.5±6.1 | - | 5.8±4.0 | 10.3±6.6 | 0.001 |
| Myocardial hemorrhage, % infarct size | 26.9±15.2 | - | 27.2±18.4 | 26.2±12.8 | 0.684 |
| *Myocardial T2*, T1 and T2 values at baseline* |  |  |  |  |  |
| T2* remote, ms | 31.5±2.4 | 31.5±2.6 | 31.8±2.5 | 31.2±1.7 | 0.218 |
| T2* infarct, ms | 32.4±7.6 | 36.4±5.7 | 26.1±5.5 | 24.8±4.1 | 0.271 |
| T2* core, ms | 14.2±3.6 | - | 15.0±3.1 | 13.7±3.9 | 0.114 |
| T1 remote (all subjects), ms | 959±25 | 959±25 | 956±24 | 963±26 | 0.278 |
| T1 infarct, ms | 1098±51 | 1104±50 | 1084±50 | 1087±55 | 0.802 |
| T1 core present, n (%) | 112 (55) | 44 (34) | 27 (90) | 41 (93) | 0.681 |
| T1 infarct core, ms | 995±57 | 1011±53 | 984±45 | 985±64 | 0.976 |
| T2 remote, ms | 49.9±2.1 | 49.9±2.1 | 49.8±2.2 | 49.8±2.1 | 0.898 |
| T2 infarct, ms | 66.8±6.0 | 65.8±5.9 | 67.7±5.4 | 69.1±6.3 | 0.354 |
| T2 core present, n (%) | 127 (63) | 53 (41) | 30 (100) | 44 (100) | - |
| T2 core, ms | 54.2±4.8 | 55.1±4.4 | 52.7±4.5 | 54.1±5.2 | 0.235 |
| *Myocardial ECV values at baseline (n=109)* |  |  |  |  |  |
| ECV remote (all subjects), % | 25.5±3.0 | 25.8±3.3 | 25.1±2.7 | 24.9±2.3 | 0.741 |
| Men | 25.1±2.9 | 25.4±3.3 | 25.2±2.8 | 24.5±2.1 | 0.405 |
| Women | 27.1±2.7 | 27.1±2.9 | 24.7 | 27.8±2.1 | 0.321 |
| ECV infarct, % | 55.5±11.4 | 52.7±12.4 | 58.6±7.3 | 60.4±8.4 | 0.502 |
| ECV hypointense infarct core, % | 42.0±11.3 | 48.7±14.8 | 40.3±6.9 | 38.9±9.4 | 0.658 |
| *CMR findings 6 months post-MI (n=211)* |  |  |  |  |  |
| LV ejection fraction at 6 months, % | 62±10 | 65±7 | 60±7 | 53±11 | 0.001 |
| LV end-diastolic volume at 6 months, ml |  |  |  |  |  |
| Men | 169±43 | 155±29 | 175±28 | 200±59 | 0.060 |
| Women | 128±24 | 127±24 | 133±30 | 133±14 | 0.977 |
| LV end-systolic volume at 6 months, ml |  |  |  |  |  |
| Men | 68±36 | 55±21 | 70±21 | 98±53 | 0.005 |
| Women | 48±17 | 43±15 | 55±11 | 75±14 | 0.045 |
| Adverse remodeling, n (%) | 23 (12) | 10 (8) | 2 (7) | 11 (26) | 0.061 |
| *Infarct characteristics at 6 months* |  |  |  |  |  |
| Infarct size, % LV mass | 13±10 | 9±8 | 16±7 | 24±10 | <0.001 |
| Myocardial iron, % LV mass | 2.4±2.2 | - | - | 2.4±2.2 | - |
| Myocardial iron, % infarct size | 10.6±9.4 | - | - | 10.6±9.4 | - |
| *Myocardial T2*, T1 and T2 values at 6 months* |  |  |  |  |  |
| T2* remote at 6 months, ms | 31.7±4.1 | 31.6±4.3 | 32.6±3.8 | 31.5±3.4 | 0.206 |
| T2* infarct at 6 months, ms | 25.7±4.4 | 27.1±4.0 | 27.0±4.3 | 21.6±2.7 | <0.001 |
| T2* core at 6 months, ms | 16.6±2.1 | - | - | 16.6±2.1 | - |
| T1 remote (all subjects), ms | 959±28 | 956±27 | 965±32 | 964±29 | 0.923 |
| T1 infarct, ms | 1054±83 | 1052±63 | 1075±55 | 1044±83 | 0.059 |
| T2 remote at 6 months, ms | 49.9±2.3 | 49.6±2.3 | 49.8±2.5 | 50.8±2.0 | 0.055 |
| T2 infarct at 6 months, ms | 56.9±4.4 | 55.9±3.7 | 57.5±3.8 | 59.5±5.5 | 0.080 |
| *Myocardial ECV values at 6 months (n=155)* |  |  |  |  |  |
| ECV remote at 6 months (all subjects), % | 25.8±2.7 | 25.4±2.6 | 25.8±2.3 | 26.6±3.1 | 0.300 |
| Men | 25.5±2.7 | 24.9±2.5 | 25.9±2.2 | 26.3±3.0 | 0.660 |
| Women | 27.0±2.5 | 26.8±2.3 | 22.9 | 30.2±1.4 | 0.043 |
| ECV infarct at 6 months, % | 51.8±11.4 | 46.7±10.9 | 57.7±5.0 | 61.1±7.4 | 0.066 |

Footnote: Abbreviations: CMR = cardiac magnetic resonance, LV = left ventricle, MI = myocardial infarction, T1 = longitudinal relaxation time, T2 = transverse relaxation time. Myocardial edema was measured with T2-mapping.

Data are given as n (%) or mean±SD. *P-values were obtained from t-test or Fisher’s test for comparisons between groups with resolved and persistent iron. †Percentage of patients with hemorrhage at 2 days (n=74).

**Supplementary Table 3.** Multivariable associations with iron status (no acute hemorrhage, resolved, persisting) (n=203) at 6 months post-STEMI in ordinal logistic regression analysis. Only statistically significant variables are reported.

| Multivariable associations | odds ratio (95% CI) | p value |
| --- | --- | --- |
| *Patient characteristics and angiographic data* | | |
| Heart rate, bpm | 1.02 (1.00, 1.04) | 0.045 |
| Male sex | 5.20 (2.05, 13.21) | 0.001 |
| Current smoker | 3.90 (1.73, 8.78) | 0.001 |
| No ST-segment resolution | 3.61 (1.54, 8.49) | 0.003 |
| TIMI flow grade 0/1 pre-PCI | 6.15 (1.77, 7.70) | 0.004 |
| *Patient characteristics, angiographic data and inflammatory blood markers* | | |
| Male sex | 5.16 (1.99, 13.38) | 0.001 |
| Current smoker | 2.91 (1.26, 6.75) | 0.013 |
| No ST-segment resolution | 4.05 (1.66, 9.87) | 0.002 |
| TIMI flow grade 0/1 pre-PCI | 5.44 (1.49, 19.87) | 0.011 |
| Neutrophils, x10^9^L | 1.22 (1.10, 1.36) | 0.001 |
| *Patient characteristics, angiographic data and infarct size* | | |
| Male sex | 4.98 (1.67, 14.91) | 0.004 |
| Current smoker | 3.66 (1.42, 9.43) | 0.007 |
| Infarct size, % LV mass | 1.14 (1.10, 1.19) | <0.001 |

Footnote: Abbreviations: CI = confidence intervals, LV = left ventricle, TIMI = thrombus in myocardial infarction.

The odds ratio (95% confidence intervals) indicates odds of increasing levels of hemorrhage at 6-months given exposure to the independent variable.

Similar results were obtained when myocardial edema was included instead of infarct size.

The clinical characteristics that were included in the multivariable model with myocardial iron status at 6 months post-STEMI were age (p=0.206), culprit artery percentage stenosis (p=0.714), body mass index (BMI) (p=0.257), symptom onset to reperfusion time (p=0.448), systolic blood pressure per 10 mmHg (p=0.824), diabetes mellitus (p=0.656), previous PCI (p=0.330), previous MI (p=0.334), previous angina (p=0.502), hypertension (p=0.909), hypercholesterolemia (p=0.051), TIMI flow grade 0/1/2 post-PCI vs. TIMI flow grade 3 post-PCI (reference category) (p=0.448).

**Supplementary Table 4.** Multivariable association of the change in LV end-diastolic volume and the change in LV ejection fraction at 6 months from baseline in 203 STEMI patients. Only statistically significant variables are reported.

| Multivariable associations | coefficient (95% CI) | p value |
| --- | --- | --- |
| *Change in LV end-diastolic volume with patient characteristics, angiographic data and 6-month iron status* | | |
| Persistent iron | 21.10 (10.92, 31.27) | <0.001 |
| LV end-diastolic volume at baseline | -1.14 (-0.22, 0.06) | 0.257 |
| *Change in LV ejection fraction with patient characteristics, angiographic data and 6-month iron status* | | |
| Persistent iron | -6.47 (-9.22, -3.72) | <0.001 |
| LV ejection fraction at baseline | 0.56 (0.44, 0.68) | <0.001 |
| Culprit artery percent stenosis | -0.15 (-0.28, -0.01) | 0.030 |
| Previous MI | 8.24 (2.80, 13.67) | 0.003 |
| Hypertension | 2.83 (0.36, 5.31) | 0.025 |

Footnote: Abbreviations: CI = confidence intervals, LV = left ventricle, MI = myocardial infarction. The coefficient (95% confidence intervals) indicates the magnitude and direction of the difference in change in LV end-diastolic volume (ml) or LV ejection fraction (%) for the patient characteristic (binary or continuous).

The clinical associates that were included in the model with the change in LV end-diastolic volume at 6 months were age (p=0.672), culprit artery percentage stenosis (p=0.125), heart rate (p=0.893), BMI (p=0.475), symptom onset to reperfusion time (p=0.193), systolic blood pressure per 10mmHg (p=0.236), male sex (p=0.775), diabetes mellitus (p=0.192), previous PCI (p=0.444), previous MI (p=0.418), previous angina (p=0.734), hypertension (p=0.939), hypercholesterolemia (p=0.567), current smoker (p=0.928), no ST-segment resolution vs. partial and complete ST-segment resolution (reference category) (p=0.430), TIMI flow grade 0/1 pre-PCI vs TIMI flow grade 2/3 pre-PCI (reference category) (p=0.465), TIMI flow grade 0/1/2 post-PCI vs. TIMI flow grade 3 post-PCI (reference category) (p=0.873).

The clinical characteristics that were included in the model with the change in LV ejection fraction at 6 months were age (p=0.934), heart rate (p=0.711), BMI (p=0.860), symptom onset to reperfusion time (p=0.710), systolic blood pressure per 10mmHg (p=0.861), male sex (p=0.980), diabetes mellitus (p=0.825), previous PCI (p=0.420), previous angina (p=0.214), hypercholesterolemia (p=0.273), current smoker (p=0.181), no ST-segment resolution vs. partial and complete ST-segment resolution (reference category) (p=0.648), TIMI flow grade 0/1 pre-PCI vs TIMI flow grade 2/3 pre-PCI (reference category) (p=0.391), TIMI flow grade 0/1/2 post-PCI vs. TIMI flow grade 3 post-PCI (reference category) (p=0.884).

# Supplementary Figure Legends

**Supplement to Figure 1 Legend.** Two patients with a similar presentation of acute anterior ST-elevation myocardial infarction. Both patients were treated by percutaneous coronary intervention and with the same anti-thrombotic drugs. At the end of the procedure both patients had TIMI coronary flow grade 3 in the culprit left anterior descending artery. Contrast-enhanced CMR 2 days later showed anteroseptal infarct (*left, yellow arrows*).

1. *A patient with myocardial hemorrhage at baseline which resolves:* LV ejection fraction decreased from 77% to 71%. This patient followed an uncomplicated clinical course.
2. *A patient with myocardial hemorrhage at baseline which persists:* LV ejection fraction decreased from 49% to 37%. This patient was re-hospitalized with new-onset heart failure.

# Supplementary Figures

**Supplementary Figure 1.** CONSORT flow diagram. CMR = cardiac magnetic resonance imaging, STEMI = ST-elevation myocardial infarction.

Acute STEMI patients

assessed for eligibility

(n = 343)

No CMR with T2* map at follow-up (n = 22)

- Death (n = 3)
- Refusal (n = 14)
- Unable to contact (n = 1)
- T2* map not acquired due to intolerance of the scan (n = 4)

## Analysis

## Follow-Up

## Enrolment with informed consent

infor

CMR 6 months post-MI with evaluable T2* maps (n = 203)

## Analysis

CMR with T2* map at follow-up (n = 224)

No CMR with T2* map (n = 57)

- Claustrophobia (n = 20)
- Death (n = 2)
- Logistical reasons (n = 15)
- Contraindications to MRI (n = 3)
- Refusal (n = 3)
- T2* map not acquired due to intolerance of the scan (n = 14)

CMR with T2* map (n = 286)

T2* maps non-evaluable due to severe motion artefact (n = 40)

CMR with evaluable T2* map (n = 246)

Not included in final analysis (n = 21)

- T2* maps non-evaluable due to severe motion artefact (n = 14)
- De novo MI (n = 7)

**Supplementary Figure 2.** Infarct zone T2* and T2 values in patients with resolved hemorrhage and persisting iron within the infarct core at 2 days and 6 months post-ST-elevation myocardial infarction.

**Supplementary Figure 3.** Left ventricular (LV) end-diastolic volume and LV ejection fraction in relation to infarct core iron status at 6 months.

# References

1. O’Gara PT., Kushner FG., Ascheim DD., et al. 2013 ACCF/AHA Guideline for the Management of ST-Elevation Myocardial Infarction. Circulation 2013;127(4):e362–425. Doi: 10.1161/CIR.0b013e3182742cf6.

2. Detection and Significance of Heart Injury in ST Elevation Myocardial Infarction. - Full Text View - ClinicalTrials.gov. Available at: https://clinicaltrials.gov/ct2/show/NCT02072850. Accessed July 31, 2016.

3. King SB., Smith SC., Hirshfeld JW., et al. 2007 Focused Update of the ACC/AHA/SCAI 2005 Guideline Update for Percutaneous Coronary Intervention. Circulation 2008;117(2):261–95. Doi: 10.1161/CIRCULATIONAHA.107.188208.

4. Messroghli DR., Greiser A., Fröhlich M., Dietz R., Schulz-Menger J. Optimization and validation of a fully-integrated pulse sequence for modified look-locker inversion-recovery (MOLLI) T1 mapping of the heart. J Magn Reson Imaging 2007;26(4):1081–6. Doi: 10.1002/jmri.21119.

5. Messroghli DR., Walters K., Plein S., et al. Myocardial T1 mapping: Application to patients with acute and chronic myocardial infarction. Magn Reson Med 2007;58(1):34–40. Doi: 10.1002/mrm.21272.

6. Xue H., Guehring J., Srinivasan L., et al. Evaluation of rigid and non-rigid motion compensation of cardiac perfusion MRI. Med Image Comput Comput-Assist Interv MICCAI Int Conf Med Image Comput Comput-Assist Interv 2008;11(Pt 2):35–43.

7. Giri S., Chung Y-C., Merchant A., et al. T2 quantification for improved detection of myocardial edema. J Cardiovasc Magn Reson 2009;11:56. Doi: 10.1186/1532-429X-11-56.

8. Verhaert D., Thavendiranathan P., Giri S., et al. Direct T2 Quantification of Myocardial Edema in Acute Ischemic Injury. JACC Cardiovasc Imaging 2011;4(3):269–78. Doi: 10.1016/j.jcmg.2010.09.023.

9. Kellman P., Arai AE., McVeigh ER., Aletras AH. Phase-sensitive inversion recovery for detecting myocardial infarction using gadolinium-delayed hyperenhancement†. Magn Reson Med 2002;47(2):372–83. Doi: 10.1002/mrm.10051.

10. Moon JC., Messroghli DR., Kellman P., et al. Myocardial T1 mapping and extracellular volume quantification: a Society for Cardiovascular Magnetic Resonance (SCMR) and CMR Working Group of the European Society of Cardiology consensus statement. J Cardiovasc Magn Reson 2013;15:92. Doi: 10.1186/1532-429X-15-92.

11. Kramer CM., Barkhausen J., Flamm SD., Kim RJ., Nagel E. Standardized cardiovascular magnetic resonance (CMR) protocols 2013 update. J Cardiovasc Magn Reson 2013;15:91. Doi: 10.1186/1532-429X-15-91.

12. Flett AS., Hasleton J., Cook C., et al. Evaluation of Techniques for the Quantification of Myocardial Scar of Differing Etiology Using Cardiac Magnetic Resonance. JACC Cardiovasc Imaging 2011;4(2):150–6. Doi: 10.1016/j.jcmg.2010.11.015.

13. Ugander M., Oki AJ., Hsu L-Y., et al. Extracellular volume imaging by magnetic resonance imaging provides insights into overt and sub-clinical myocardial pathology. Eur Heart J 2012;33(10):1268–78. Doi: 10.1093/eurheartj/ehr481.

14. Arheden H., Saeed M., Higgins CB., et al. Measurement of the Distribution Volume of Gadopentetate Dimeglumine at Echo-planar MR Imaging to Quantify Myocardial Infarction: Comparison with 99mTc-DTPA Autoradiography in Rats. Radiology 1999;211(3):698–708. Doi: 10.1148/radiology.211.3.r99jn41698.

15. Medical Research Council Guidelines for good clinical practice in clinical trials. Available at: http://www.mrc.ac.uk/documents/pdf/good-clinical-practice-in-clinical-trials/. Accessed July 31, 2016.

16. Hicks KA., Tcheng JE., Bozkurt B., et al. 2014 ACC/AHA Key Data Elements and Definitions for Cardiovascular Endpoint Events in Clinical Trials: A Report of the American College of Cardiology/American Heart Association Task Force on Clinical Data Standards (Writing Committee to Develop Cardiovascular Endpoints Data Standards). J Am Coll Cardiol 2015;66(4):403–69. Doi: 10.1016/j.jacc.2014.12.018.
